# Imaging in Biologically-Relevant Environments with AFM Using Stiff qPlus Sensors

**DOI:** 10.1038/s41598-018-27608-6

**Published:** 2018-06-19

**Authors:** Korbinian Pürckhauer, Alfred J. Weymouth, Katharina Pfeffer, Lars Kullmann, Estefania Mulvihill, Michael P. Krahn, Daniel J. Müller, Franz J. Giessibl

**Affiliations:** 1 University of Regensburg, Institute of Experimental and Applied Physics, Regensburg, 93053 Germany; 2 University of Regensburg, Institute for Molecular and Cellular Anatomy, Regensburg, 93053 Germany; 3 Eidgenössische Technische Hochschule (ETH) Zürich, Department of Biosystems Science and Engineering, Basel, 4058 Switzerland; 4 University Hospital of Münster, Internal Medicine D, Münster, 48149 Germany

## Abstract

High-resolution imaging of soft biological samples with atomic force microscopy (AFM) is challenging because they must be imaged with small forces to prevent deformation. Typically, AFM of those samples is performed with soft silicon cantilevers (k ≈ 0.1–10 N/m) and optical detection in a liquid environment. We set up a new microscope that uses a stiff qPlus sensor (k ≥ 1 kN/m). Several complex biologically-relevant solutions are non-transparent, and even change their optical properties over time, such as the cell culture medium we used. While this would be problematic for AFM setups with optical detection, it is no problem for our qPlus setup which uses electrical detection. The high stiffness of the qPlus sensor allows us to use small amplitudes in frequency-modulation mode and obtain high *Q* factors even in liquid. The samples are immersed in solution in a liquid cell and long tips are used, with only the tip apex submerged. We discuss the noise terms and compare the minimal detectable signal to that of soft cantilevers. Atomic resolution of muscovite mica was achieved in various liquids: H_2_O, Tris buffer and a cell culture medium. We show images of lipid membranes in which the individual head groups are resolved.

## Introduction

Atomic force microscopy (AFM) imaging at the atomic scale in biologically-relevant conditions is challenging because of the complex interaction between tip and sample. Biological samples need to be imaged with minimal interaction forces because if the force is larger than about 100 pN the sensitive sample might get damaged^1,2^. Typically, AFM imaging of these sample systems is performed in tapping mode^3–5^ in liquid environments by scanning the sample with a soft silicon cantilever (stiffness *k* ≈ 0.1–10 N/m) that is completely immersed in liquid^6^. In tapping mode, soft cantilevers are usually used to scan biological samples as one obtains a large signal and reversible or irreversible deformations of the sample are reduced^7–9^. In this work we demonstrate that frequency modulation AFM (FM-AFM) with stiffer sensors (*k* ≥ 1 kN/m), a technique which was used to obtain submolecular resolution on organic molecules in ultra-high vacuum (UHV) at low temperature^10^ and even room temperature^11^, facilitate imaging also of biological samples with high spatial resolution. Frequency modulation AFM (FM-AFM)^12–15^ is a technique in which the sensor is driven to oscillate at a given amplitude and the measured variable is the frequency shift Δ*f* which is itself a measure of the force gradient *k*_ts_ between tip and sample. The key question in optimizing imaging is not the stiffness of the sensor but the detectable force gradient that is limited by instrumental noise.

**Figure 1:**
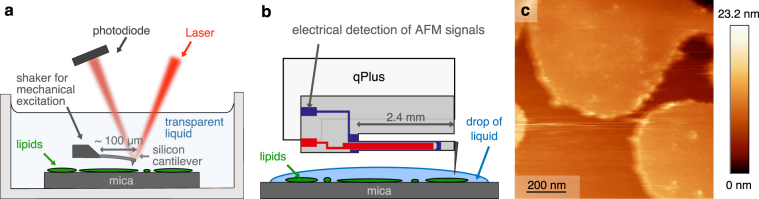
Comparison of AFM setups for imaging biological samples with silicon cantilevers and qPlus AFMs. (**a**) Schematic drawing of a typical silicon cantilever AFM setup which is completely immersed in a transparent liquid. (**b**) Schematic drawing of the qPlus AFM setup for measurements in a drop of liquid. (**c**) FM-AFM image of lipid membrane islands (extracted from *Halobacterium salinarum*) performed in z-feedback with *f*_0_ = 18.92 kHz, *k* = 3500 N/m, *A* = 150 pm and Δ*f* = +20 Hz. Buffer solution: 300 mM KCl and 20 mM Tris-HCl (pH 7.5).

In FM-AFM, noise is characterized by the minimal detectable force gradient *δ*〈*k*_ts_〉_min_^16^. It is a function of the quality factor of the cantilever, *Q*, which relates the energy stored in the cantilever to the energy lost per cycle. Operation in liquid lowers the *Q* value due to additional damping. Soft cantilevers completely immersed in liquid (see Fig. 1a) have a very low *Q* value, around 1–30^17,18^. A solution to maintain high Q values is to use a stiffer sensor like the self-sensing qPlus sensors^19,20^ (*k* ≥ 1 kN/m), shown in Fig. 1b).

In most cases, an AFM sensor is excited mechanically (also called acoustic excitation) with a vibrating piezo stack. The frequency response of the oscillation with respect to the excitation is a single excitation peak at the resonance frequency and a smooth monotonic phase curve. The monotonic phase response is exploited when performing FM-AFM as it allows a Phase Locked Loop (PLL) to follow the current resonance frequency. It has been observed that driving a soft cantilever mechanically in liquid causes many unwanted new peaks near the resonance frequency, an effect referred to as the “forest of peaks”^21^. In addition, the phase response is no longer monotonic, which prevents stable amplitude feedback in FM-AFM. To avoid the “forest of peaks” effect and a non-monotonic phase either a direct excitation method, such as magnetic excitation^22,23^ or photothermal exciation^24,25^, or a stiffer sensor is required.

The success of FM-AFM with stiff sensors and piezoelectric detection in vacuum^26–28^ demonstrates the advantages of using small amplitudes (<100 pm) because they yield higher sensitivity to short range forces^29^. These small amplitudes on the order of the decay length of the short-range interaction forces cannot be realized with a soft cantilever because of the well-known “jump-to-contact” problem^20^.

Scanning in ambient conditions where the sample is covered with a hydration layer^30^ was a first step towards imaging in a biologically-relevant environment. Optimizing imaging with the qPlus sensor in ambient conditions led to reproducible images with atomic resolution on various sample systems^31–33^ and sensor oscillation modes^34^. In order to image with a qPlus sensor in liquid environments, specifically with conducting liquids, electric detection of the AFM signal demands that either only the tip is submerged or the sensor is covered by an insulation layer. As the second method could lower the *Q*-value, we pursued the first option. Previous measurements were performed with qPlus sensors in aqueous solutions done with adding just a drop of liquid on the sample^16^. Ichii *et al*.^35^ showed that imaging of muscovite mica at the atomic scale was possible with qPlus sensors in such a drop. In Fig. 1c we show large lipid bilayer patches that are non-destructively imaged in a drop of buffer solution (~20 μl) with qPlus sensors using FM-AFM. As the small liquid volume evaporated within about 15 min., high resolution imaging was impractical.

**Figure 2:**
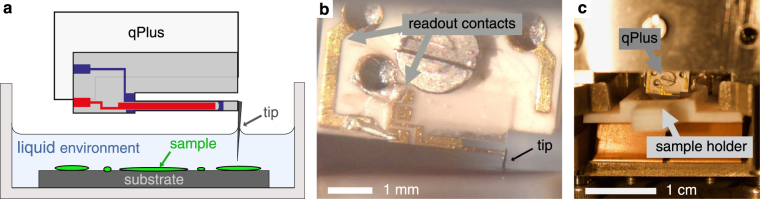
qPlus AFM setup for liquid measurements. (**a**) Schematic drawing of the qPlus AFM setup for measurements in a liquid bath. The sample is immersed in liquid and only the tip apex is submerged. (**b**) View on a qPlus sensor (equipped with a long sapphire tip) oscillating in liquid. (**c**) Image of the AFM head including the sample holder with integrated liquid cell.

Our goal is to achieve high spatial resolution of soft biological samples. We therefore moved from this setup which allowed us to demonstrate proof of principle to a more controlled environment that would allow us to achieve high spatial resolution. A sample holder with an integrated liquid bath (*A*_cell_ = 85 mm^2^) was created (Fig. 2). The new sample holder allows us to use up to 420 μl of solution. As a consequence, very long tips (~500–1000 μm) had to be glued to the sensor’s oscillating prong. The qPlus sensor with the tip submerged in liquid is shown in Fig. 2b and the AFM setup including the liquid cell is depicted in Fig. 2c. This setup allows us to use any kind of tip material. For this study, we used sapphire which is very hard and chemically inert. With this new setup, we are able to image biological samples non-destructively. We show images of a lipid layer extracted directly from a biological sample in which the individual lipid head groups can be observed.

## Results and Discussion

**Figure 3:**
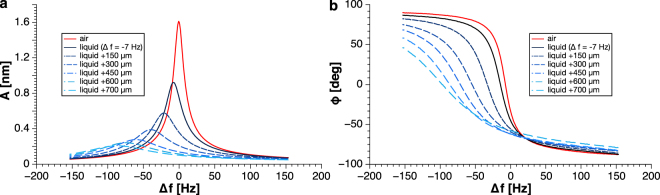
Resonance behaviour of qPlus sensor as a function of penetration depth. (**a**) Resonance curves in different penetration depths shown in a blue color-code and the reference one in air shown in red. The associated phases are depicted in (**b**) with the same color scheme and obviously it stays monotonic for all the time. The representative sensor had a resonance frequency of *f*_0_ = 23.41 kHz and stiffness *k* = 1800 N/m in air.

We first evaluated the influence of the penetration depth of the tip in liquid on the qPlus resonance curve. To do so, we recorded a resonance curve of the mechanically excited sensor in air and approached to the water layer. The distance where the tip starts penetrating the liquid can be seen by a shift in resonance frequency. To ensure that the tip is vibrating in liquid we defined Δ*f* = −7 Hz as our first reference point in liquid and recorded resonance spectra at various penetration depths by approaching the qPlus sensor to the support using the coarse motor. The drive amplitude was kept constant for all data. The data shown in Fig. 3a display a continuous decrease of the oscillation amplitude at resonance starting from about 1.6 nm in air until it levels off at around 12.5% of its original value. The largest achievable penetration depth is limited by the length of the tip (representative sensor had a 700 μm long sapphire tip). It is important that the phase stays monotonic for all penetration depths as verified in Fig. 3b.

In FM-AFM, noise is characterized by the minimal detectable force gradient *δ*〈*k*_ts_〉_min_, composed by detector $\delta {k}_{{{\rm{ts}}}_{{\rm{\det }}}}$, thermal $\delta {k}_{{{\rm{ts}}}_{{\rm{th}}}}$ and oscillator noise $\delta {k}_{{{\rm{ts}}}_{{\rm{osc}}}}$^36^. As these noise sources are statistically independent, *δ*〈*k*_ts_〉_min_ can be written as^16^

1 $$\delta {\langle {k}_{{\rm{ts}}}\rangle }_{{\rm{\min }}}=\sqrt{\delta {k}_{{{\rm{ts}}}_{{\rm{th}}}}^{2}+\delta {k}_{{{\rm{ts}}}_{{\rm{\det }}}}^{2}+\delta {k}_{{{\rm{ts}}}_{{\rm{osc}}}}^{2}}$$

The individual noise terms are given by^16^

2 $$\delta {k}_{{{\rm{ts}}}_{{\rm{th}}}}=\sqrt{\frac{4k{k}_{B}TB}{\pi {f}_{0}{A}^{2}Q}}\propto \sqrt{\frac{1}{Q}}$$

3 $$\delta {k}_{{{\rm{ts}}}_{{\rm{\det }}}}=\sqrt{\frac{8}{3}}\frac{k{n}_{q}{B}^{\frac{3}{2}}}{{f}_{0}A}$$

4 $$\delta {k}_{{{\rm{ts}}}_{{\rm{osc}}}}=\frac{k{n}_{q}\sqrt{2B}}{AQ}\propto \frac{1}{Q}$$

The above formulae include quality factor *Q*, the stiffness *k*, the Boltzmann constant *k*_*B*_, the temperature *T*, the bandwidth *B*, the resonance frequency *f*_0_, the amplitude *A* and the deflection noise density *n*_*q*_. Thermal and oscillator noise depend on the quality factor *Q*, which is related to the inverse loss of energy per cycle Δ*E*_cycle_ relative to the stored energy in the resonator *E* by *Q* = *E*/(Δ*E*_cycle_⋅2*π*).

**Figure 4:**
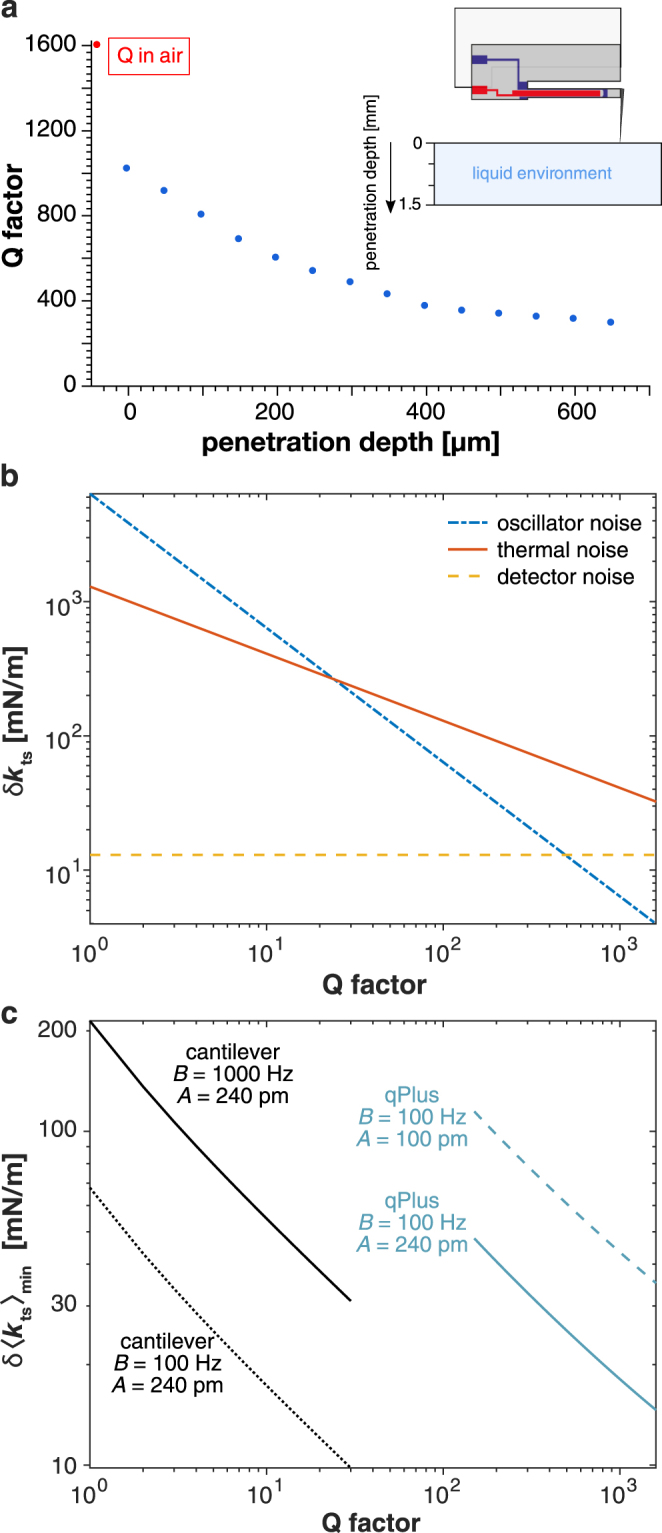
Analysing *Q* dependence of FM-AFM noise. (**a**) Dependence of the *Q* factor on the tip’s penetration depth together with a schematic drawing. The reference *Q* value in air (1597) is marked with a red dot. (**b**) Thermal noise *δk*_ts,th_ (solid, red), detector noise *δk*_ts,det_ (dashed, yellow) and oscillator noise *δk*_ts,osc_ (dashed-dotted, blue) of qPlus sensor plotted over *Q*. (**c**) Minimal detectable force gradient *δ*〈*k*_ts_〉_min_ plotted vs. *Q* factor of silicon cantilever (*A* = 240 pm, colored black) with both a common *B* = 1 kHz and a small *B* = 100 Hz bandwidth compared to qPlus (colored blue, *B* = 100 Hz, *A* = 100 pm, dashed, and *A* = 240 pm, solid).

We calculated the *Q* factor from the slopes of the penetration depth dependent phases at *f*_0_. The depth dependence of the *Q* factor is plotted in Fig. 4a with the value in air marked in red as reference. There was a continuous decrease from 1600 in air until it leveled off at approximately 300. *Q* values up to 1000 in liquid are possible if the penetration depth is small enough. The data were reproducible for a variety of stiffnesses (ranging from 1800 to 8300 N/m), different tip geometries and even for thermal excitation of the sensor.

We also characterized the evaporation rate of water from the cell. The rate was 0.39 μl/min for three different cells at a humidity of 40%. Assuming a measurement time of 30 min for a large overview scan, this results in a change of 138 μl. For a penetration depth of 600 μm, the *Q* factor will change from 300 to 350.

Figure 4b shows a calculation of the individual noise terms $\delta {k}_{{{\rm{ts}}}_{{\rm{th}}}}$, $\delta {k}_{{{\rm{ts}}}_{{\rm{\det }}}}$ and $\delta {k}_{{{\rm{ts}}}_{{\rm{osc}}}}$ from Eqs (2–4) for a qPlus sensor as a function of *Q*. The parameters of a new custom qPlus sensor with higher *f*_0_ used for the calculation can be found in Table 1. The curves show that for the given setup the thermal noise $\delta {k}_{{{\rm{ts}}}_{{\rm{th}}}}$ dominates. Oscillator noise $\delta {k}_{{{\rm{ts}}}_{{\rm{osc}}}}$ becomes significant for *Q* values below 200 but is not as important as for soft silicon cantilevers where *Q* is in the range of 1 up to 30. The detector noise $\delta {k}_{{{\rm{ts}}}_{{\rm{\det }}}}$ is independent of *Q* and therefore is dominated by the other noise terms for *Q* values below 200. The noise terms in Eqs (2–4) show that sensor properties *k* and *f*_0_ are also important in addition to *Q*. Silicon cantilevers have a higher *f*_0_ and lower *k* than qPlus sensors which leads to an advantage in *k*/*f*_0_ ratio. In liquid environments, the total force gradient noise is heavily influenced by the low *Q*. We compared the total noise in liquid to that from a standard cantilever by plotting the qPlus total noise over the *Q* range of 150 to 1600 and the cantilever noise over *Q* from 1 to 30 in Fig. 4c. The parameters for a cantilever taken from Fukuma *et al*.^14^ and a qPlus sensor used for the calculations can be found in Table 1. As the detector noise of qPlus is larger, the scanning speed has to be significantly slower to obtain a similar signal-to-noise ratio since the detector noise increases proportional to *B*^3/2^ in FM-AFM as shown in Eq. (3). Therefore tapping mode with soft silicon cantilevers is typically still faster than FM-AFM imaging using the qPlus sensor.

**Table 1:** Comparison of sensor and scan parameters which are influencing the total minimal detectable force gradient.

Parameter	qPlus	Cantilever
*f*_0_ [kHz]	56.59	136
*k* $[\frac{{\rm{N}}}{{\rm{m}}}]$	1800	42
*A* [pm]	100	240
*n*_*q*_ $[\text{fm}/\sqrt{{\rm{Hz}}}]$	25	17
*B* [Hz]	100	1000
*Q*	150–1600	1–30

The values for the cantilever are taken from Fukuma *et al*.^14^ and the ones for qPlus are typical specifications of our latest custom qPlus sensors which have a higher resonance frequency (as described in^56^).

**Figure 5:**
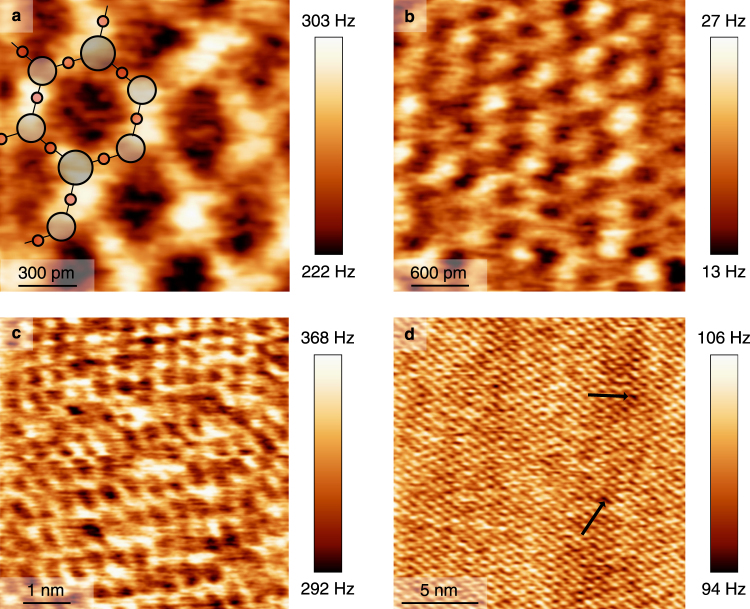
FM-AFM images in quasi-constant height mode (defined in the text) of muscovite mica in different environments. (**a**) Image taken in ambient conditions with Δ*f* = +257 Hz and *A* = 78 pm. A possible lattice configuration of muscovite mica is overlaid. (**b**) Image taken in H_2_O with Δ*f* = +20 Hz and *A* = 210 pm. (**c**) Image taken in HAM culture medium with Δ*f* = +330 Hz and *A* = 112 pm. (**d**) Image taken in Tris-HCl buffer with Δ*f* = +100 Hz and *A* = 100 pm.

Imaging was performed in various environments to evaluate the possibility of imaging at the atomic scale in biologically relevant environments. Our measurements were performed on muscovite mica KAl_2_(OH,F)_2_(AlSi_3_O_10_) which is a well known substrate for immobilizing biological samples like lipids^37–39^ or membrane proteins^40–42^. As biological environments we used ultrapure water, Tris buffer and a non-transparent cell culture medium. As a reference, Fig. 5a shows atomic resolution with overlaid lattice in air. The periodicity was found to be 0.52 nm which fits to the literature value^43^.

For the measurements in ultra-pure (18 MΩ/cm) water we added 60 μl onto the freshly cleaved muscovite mica. The sensor’s resonance frequency was determined to be 23.3 kHz in air and the *Q* value was 1777 in air (*k* = 1800 N/m). The *Q* value in liquid was 714 near sample contact. The frequency shift image with atomic resolution depicted in Fig. 5b was recorded with an amplitude of 210 pm (best signal-to-noise ratio in this case) in quasi-constant height mode. This means that the feedback loop of the height control was set very slow to merely compensate the drift.

We then acquired images in a cell culture medium called HAM (see materials and methods) as shown in Fig. 5c. For imaging in cell culture medium we used a qPlus sensor with *k* = 1800 N/m, *f*_0_ = 56.59 kHz and a *Q* of 1954 in air. We scanned in 100 μl cell culture medium and *Q* decreased to 147 at close distance to the sample in liquid. The frequency shift image Fig. 5c, where again the honeycomb lattice could be imaged, was recorded with an amplitude of 112 pm in quasi-constant height mode.

Finally we added 200 μl 10 mM Tris-HCl buffer solution (pH 8.0) onto freshly cleaved muscovite mica and used the same qPlus as for the H_2_O measurement. The *Q* value was 275 during the measurement. The 20 nm by 20 nm frame pictured in Fig. 5d was measured in quasi-constant height with 100 pm amplitude and shows atomic resolution with naturally occuring defects marked by black arrows.

**Figure 6:**
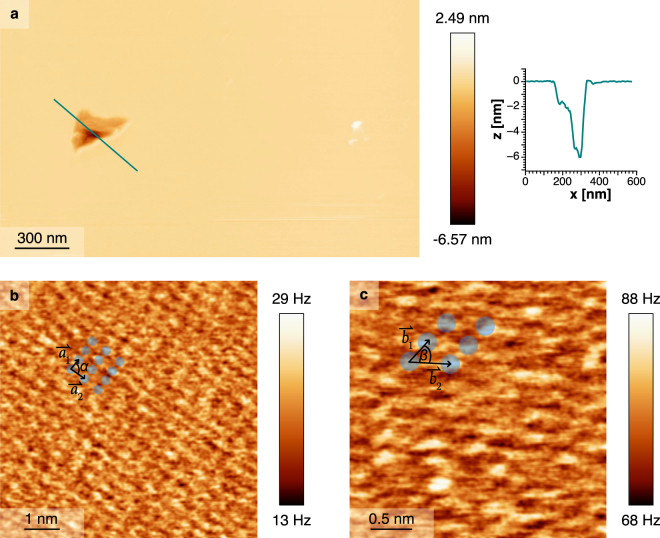
FM-AFM images of a lipid bilayer adsorbed on mica and measured in buffer solution. (**a**) Topography overview scan of the lipid bilayer with a large artificially created defect and an additional line profile along the cyan colored line is included. The imaging parameters were *A* = 200 pm and Δ*f* = +25 Hz. (**b**) High resolution image of the lipid heads in Δ*f* feedback taken in quasi-constant height mode with *A* = 100 pm and Δ*f* = +20 Hz. The sensor’s resonance frequency *f*_0_ was 15.57 kHz. The rectangular lattice (*α* = 90°) is indicated by the headgroup positions marked by blue dots with lattice vectors ${\overrightarrow{a}}_{1}$ and ${\overrightarrow{a}}_{2}$. (**c**) Image of the lipid heads in Δ*f* feedback taken in quasi-constant height mode with *A* = 106 pm and Δ*f* = +76 Hz. The sensor’s resonance frequency *f*_0_ was 15.72 kHz. The headgroup positions marked by blue dots indicate an oblique lattice. The angle *β* between the lattice vectors ${\overrightarrow{b}}_{1}$ and ${\overrightarrow{b}}_{2}$ was 64°.

The new setup allowed us to study phospholipid bilayers with longer measurement time (~3–4 h at 50% humidity). Therefore, we adsorbed liposomes (made from egg-PC) onto a freshly cleaved mica sheet which formed lipid bilayers^37,38,44,45^. Imaging was performed in approximately 250 μl buffer solution. Figure 6a shows an overview scan where we artificially created a hole into the lipid bilayer by approaching 10 nm from a Δ*f* = +25 Hz setpoint towards the sample and scratching along a 100 × 100 nm large frame. By analyzing the line profile over the scratched hole (Fig. 6a), one can see that the hole is ~5.3 nm deep which agrees to the height of a lipid bilayer^46^. Furthermore, there is a ~1.5 nm step in the line profile visible that might refer to lipid headgroups having been bent over by the scratching. It is important to note that imaging with the stiff qPlus sensor was non-destructive since the image shows no further manipulated lipid layer than the artificially created defect.

In Fig. 6b we scanned the lipids in a quasi-constant height mode to image the lipid headgroups with high spatial resolution. The image presents a rectangular lattice with lattice vectors ${\overrightarrow{a}}_{1}$ and ${\overrightarrow{a}}_{2}$ having magnitudes 0.34 nm and 0.45 nm shown in Fig. 6b. When scanning a second sample (liposomes extracted from the same sample) we observed an oblique lattice with lattice vectors ${\overrightarrow{b}}_{1}$ and ${\overrightarrow{b}}_{2}$ having magnitudes 0.26 nm and 0.55 nm and angle *β* between them of 64°, shown in Fig. 6c. The two structures can be explained by the various types of lipids (including different fatty acid distributions) which are present in natural lipid samples of Egg-PC. The area per lipid head can be calculated with the measured lattice vectors and was found to be 0.15 nm^2^ for the rectangular lattice and 0.13 nm^2^ for the oblique lattice.

These areal densities agree with previously quoted values (see, e.g. Chapter 10 in ref.^47^), however they are significantly smaller than those values quoted by X-Ray diffraction studies^46,48^. This might be because AFM can identify the individual domains of the lipid reconstruction, whereas X-Ray diffraction yields only an ensemble average.

**Figure 7:**
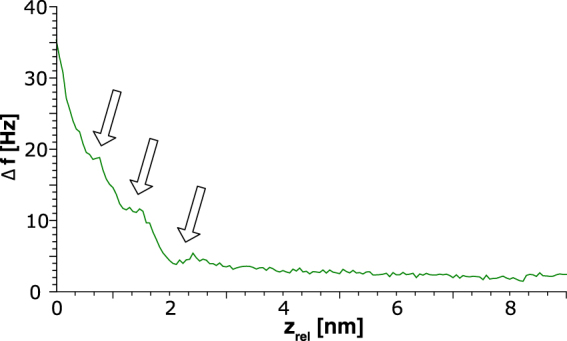
Frequency shift-distance spectrum on Egg-PC lipid bilayer. Arrows indicate peaks in the spectrum, discussed in detail in the text.

As well as images of the Egg-PC lipid bilayer, we collected Δ*f*(*z*) spectroscopy. One such spectrum is shown in Fig. 7, in which we can observe plateaus in the Δ*f*(*z*) curve. Asakawa and co-workers acquired FM-AFM data of a model bilayer system (dipalmitoylphosphatidylcholine) in which they were able to observe similar features^45^. They associated these features with ordered hydration layers. Previous work with the qPlus sensor has demonstrated that hydration layers can be observed above the calcite surface^33^.

## Conclusion

We have demonstrated that it is possible to image soft biological samples in liquid using stiff qPlus sensors. The qPlus sensors can be equipped with various kinds of tips such as metal tips that are used in tip enhanced Raman spectroscopy (TERS)^49,50^ and scanning near field optical microscopy (SNOM)^51,52^. Furthermore FM-AFM with a PLL works without any further system adjustments due to the monotonic phase around *f*_0_ in air and liquid. We showed that stiffer sensors like the qPlus can maintain high *Q* values greater than 150 in liquid. The *Q* factor plays a role for the minimal detectable force gradient *δ*〈*k*_ts_〉_min_ and we note that silicon cantilevers suffer more from a liquid environment than qPlus sensors do. The comparison of *δ*〈*k*_ts_〉_min_ for the two sensor types showed comparable values with the qPlus sensors. Challenges remain in improving imaging speed. With our liquid qPlus setup, atomic resolution on mica was achieved with amplitudes around one Angstrom in a variety of liquids including a non-transparent (pink) cell culture medium. These small amplitudes, with their high sensitivity to short-range forces, have been used to acquire images in vacuum environments with unprecedented spatial resolution. We hope that our future work will exploit this advantage and allow us to finally observe complex biological samples with atomic resolution in liquid environments. The development of a liquid bath allowed us to measure in a more controlled environment with longer measurement time. With it, we were able to image the lipid heads of a lipid bilayer adsorbed onto mica.

## Materials and Methods

### AFM setup

Our setup is a custom-design AFM head based on the Pan design^53^ with a vertical approach and small mechanical loop for high stability and low thermal drift. Moreover the electrical wiring of the microscope was optimized including the use of a better operational amplifier design^54^ to lower the deflection noise density *n*_*q*_ of the system from 35 fm/√Hz (ref.^16^) down to 25 fm/√Hz. Imaging was performed with qPlus sensors equipped with sharp sapphire tips which were created by smashing a sapphire crystal with a hammer and gluing an appropriate splinter to the end of the prong.

### Samples

For imaging we glued a thin muscovite mica (V1 grade purchased from Plano GmbH) disk to the bottom of the liquid cell, which was made out of Teflon, and cleaved it with mechanical exfoliation. Our results show that this leads to homogenous flat surfaces. For measurements in liquids we filled the liquid bath with the appropriate solution right after cleaving.

The cell culture medium we used was HAM medium, which is non-transparent (pink color). It contains salts (NaHCO_3_, CaSO_4_, Na_2_HPO_4_, CaCl_2_, KCl, NaCl), a mineral solution, Resazurin, Na_2_S and a small fraction of other solutes.

For Fig. 1b, lipid membranes were extracted together with purple membranes from *Halobacterium salinarum*^55^. For adsorption, 2 μl of the extracted lipid mixture was diluted in 120 μl adsorption buffer (300 mM NaCl, 20 mM Tris, pH 7.5) and finally a drop (~20 μl) of this solution was incubated on muscovite mica for 30 minutes. For imaging, the adsorption buffer was exchanged with imaging buffer (150 mM NaCl, 20 mM Tris, pH 7.5) and the sample was washed five times to remove unattached lipids.

For Fig. 6, liposomes were prepared in adsorption buffer (300 mM NaCl, 25 mM HEPES, pH 7.4) by extruding 25 mg/ml lipids (L-*α*-phosphatidylcholine from egg yolk, Egg-PC #840051, Avanti Polar Lipids) through a 0.1 μm polycarbonate membrane using a Mini-Extruder (Avanti Polar Lipids, Inc.). For adsorption, liposomes were diluted to 25 μg/ml in adsorption buffer and incubated on fleshly cleaved mica for 30 min. After this, the adsorption buffer was exchanged with imaging buffer (150 mM NaCl, 25 mM HEPES, pH 7.4) and washed five times to remove unattached lipids.

### Data availability

The datasets generated during and analysed during the current study are available from the corresponding author on reasonable request.
